# Dioxin-like activities in serum across European and Inuit populations

**DOI:** 10.1186/1476-069X-5-14

**Published:** 2006-05-25

**Authors:** Manhai Long, Birgitte S Andersen, Christian H Lindh, Lars Hagmar, Aleksander Giwercman, Gian-Carlo Manicardi, Davide Bizzaro, Marcello Spanò, Gunnar Toft, Henning S Pedersen, Valentyna Zvyezday, Jens Peter Bonde, Eva C Bonefeld-Jorgensen

**Affiliations:** 1 Unit of Cellular & Molecular Toxicology, Department of Environmental and Occupational Medicine, Institute of Public Health, University of Aarhus, Vennelyst Boulevard 6, DK-8000 Aarhus C, Denmark; 2 Department of Occupational and Environmental Medicine and Psychiatric Epidemiology, University Hospital, SE-221 Lund, Sweden; 3 Fertility Centre, Malmö University Hospital, Lund University, Malmö, SE-205 02, Sweden; 4 Laboratory of Genetics, Department of Agricultural Sciences, University of Modena and Reggio Emilia, Viele Kennedy 17 – Reggio Emilia I-41100 Modena, Italy; 5 Institute of Biology and Genetics, Politechnical University of Marche, Via Brecce Bianche 1-60131 Ancona, Italy; 6 Section of Toxicology and Biomedical Sciences, BIOTEC-MED, ENEA Casaccia, Via Anguillarese 301, 00060 Rome, Italy; 7 Department of Occupational Medicine, Aarhus University Hospital, Nørrebrogade 44, build. 2C, DK-8000 Aarhus C, Denmark; 8 Center for Arctic Environmental Medicine, postbox 570DK-3900 Nuuk, Greenland, Denmark; 9 Laboratory of Human Reproduction, Kharkiv State Medical University, Klochkovskaya Street 156-A, room 14, 61145 Kharkiv, Ukraine

## Abstract

**Background:**

Persistent organic pollutants (POPs) such as polychlorinated dibenzo-*p*-dioxins/furans, polychlorinated biphenyls (PCBs) and organochlorine pesticides can cause a series of adverse effects on e.g. reproduction in animals and humans, many of which involve the aryl hydrocarbon receptor (AhR). The aim of the present study was to compare the integrated serum level of AhR mediated activity among European and Inuit populations, and evaluate whether the activity was associated to the selected POP markers, 2,2',4,4',5,5'-hexachlorobiphenyl (CB-153) and 1,1-dichloro-2,2-bis(p-chlorophenyl)-ethylene (*p,p'*-DDE).

**Methods:**

The study included 338 males from Greenland (Inuit's), Sweden, Warsaw (Poland) and Kharkiv (Ukraine). The AhR transactivity of serum extracts alone (AhRag) and competitive AhR activity (AhRcomp) upon co-exposure with 2,3,7,8-tetrachlorodibenzo-*p*-dioxin (TCDD) were determined in the lipophilic serum fraction containing the POPs using the AhR mediated luciferase reporter Hepa1.12cR cell assay.

**Results:**

The European groups showed higher median level of AhR-TEQ (TCDD toxic equivalents) compared to the Inuit's, whereas higher incidence of Inuits sample further induced AhRcomp activity. Neither AhRag nor AhR-TEQ were correlated to CB-153 or *p,p'*-DDE for any of the study groups. Multiple regressions showed a significant heterogeneity of association between the CB-153 and the AhRcomp across the study groups, and accordingly a negative association between AhRcomp and CB-153 was found for the Kharkiv group.

**Conclusion:**

No consistent correlation between AhR activities and two POP markers was found. Although the difference of AhRag between European and Inuit men could not be explained by CB-153 or *p,p'*-DDE levels alone, we believe that the variation of AhR serum activity reflects different pattern of POP exposure, genetics and/or life style factors.

## 1. Background

The polychlorinated dibenzo-*p*-dioxins/furans (PCDDs/PCDFs), polychlorinated biphenyls (PCBs) and organochlorine pesticides, such as 2,2-bis(p-chlorophenyl)-1,1,1-trichloroethane (DDT), are prominent among the persistent organic pollutants (POPs). Owing to their negative effects on wildlife and human health, PCBs and DDT were restricted or totally banned in most countries during the 1970s. However, PCBs can still be released into the environment from poorly maintained hazardous waste site and illegal or improper dumping of PCB wastes like leaking from old electrical transformers, and DDT is still used in some developing countries [1]. Being resistant to both biotic and abiotic degradation, DDT (mainly as its major metabolite, 1,1-dichloro-2.2-bis (p-chlorophenyl)-ethylene (*p,p'*-DDE)) and PCBs bioaccumulate and magnify in animals and humans [2,3]. Residues have been detected in various food substances and in human adipose tissue, milk, and serum [1,4]. While PCBs and DDT contamination is ubiquitous globally, a high intake of fish and sea mammal food in the Arctic regions is associated with extraordinary high exposure [5,6]. Also Swedish fisherman's families of the Baltic Sea with a high consumption of herring and salmon being contaminated with POPs, constitute a highly exposed group [7,8]. For the general populations in Eastern Europe, the burden of POPs has been less systematically examined [9,10].

It has been documented that exposure to POPs such as dioxins (e.g. 2,3,7,8-tetrachlorodibenzo-*p*-dioxin, TCDD) and dioxin-like compounds (DLCs) such as *non-ortho *and *mono-ortho *PCBs may cause a series of negative effects both in animal experiments and in human epidemiologic studies including carcinogenicity [11], immunotoxicity and adverse effects on reproductive, neurobehavioral [12]. The toxicity of dioxins and DLCs is mediated mainly through binding to the aryl hydrocarbon receptor (AhR), which is an intracellular ligand-dependent transcriptional factor expressed in most tissues of mammals [13]. Upon receptor-ligand binding and translocation to the nucleus, the complex with the AhR nuclear translocator binds to the DNA dioxin-responsive elements, causing induction of gene transcription, for instance, encoding for metabolic enzymes [14]. More recently, interference of POPs or their metabolites with hormone receptors has also been observed [15,16]. Previous studies demonstrated the presence of a two-way cross talk between the intracellular signalling pathways involving the estrogen- (ER), androgen- (AR) and the Ah- receptor [17]. Several studies on wildlife and laboratory animals showed that exposure to PCBs and *p,p'*-DDE can affect reproductive and endocrine functions [5,18]. However, human epidemiologic data are limited and major gaps in knowledge continue to preclude evidence based risk assessment.

Since dioxins and DLCs exist as complex mixtures of various congeners throughout the environment, the concept of TEQ (TCDD toxic equivalent) has been introduced to simplify risk assessment and regulatory control [3]. The classical TEQs are calculated by multiplying the concentration of individual PCDDs/PCDFs/PCBs by their respective Toxic Equivalency Factors (TEFs), which correspond to the relative potency of the congener to generate AhR-mediated effects in relation to TCDD, the most potent AhR ligand. Previous studies emphasize that assessment of the toxicological potential of a chemical mixture is much more complex than can be deduced by a given calculated TEQ value [5,19]. There are several drawbacks using the TEF concept for risk assessment of mixtures of POPs such as expensive and time consuming gas chromatography mass spectrometry (GC-MS) determinations, small concentrations of individual congeners, presence of compounds not routinely measured or unknown substances with AhR affinity, the lack of TEF values for several POPs, and possible antagonistic or synergistic interactions between POPs [20-22]. Thus there is a need for an integrated risk assessment of dioxins and DLCs. The *in vitro *AhR mediated chemical activated luciferase gene expression (CALUX) bioassay has proven to be a quick and sensitive assay to detect the AhR mediated potential of pure chemicals [20-23], extracts of environmental and biological matrices and thus the integrated TEQ value (CALUX-TEQ) of complex mixtures as found in sediment, pore water, bovine and human milk, human serum and follicular fluid [24].

This study was a part of the EU supported research project Inuedo [25] with the main objective to elucidate the fertility in European and Inuit groups with different intake of POPs [26,27]. The 2,2',4,4',5,5'-hexachlorobiphenyl (CB-153) and *p,p'*-DDE were selected as proxy biomarkers of POPs exposure because CB-153 generally correlates with serum total PCB concentration and chemical derived TEQ [28,29], and *p,p'*-DDE was considered as a relevant marker of POPs [30]. The specific aim of the present study was to compare the actual level of AhR mediated dioxin-like activity in the lipophilic serum fraction between European and Inuit study groups, and to evaluate whether the tested dioxin-like activity was correlated to CB-153 or *p,p'*-DDE.

## 2. Methods

### 2. 1. Study groups and sampling

The Inuedo source populations encompassed women and their male spouses who had antenatal care visits from May 2002 through February 2004 at the local hospitals in Greenland, Warsaw, Poland and Kharkiv, Ukraine [26]. An established cohort of Swedish fishermen was also included [31]. The study was approved by the local ethical committees representing all participating populations and all subjects signed an informed consent. The subjects of the present study were adult males randomly selected from the source populations. Demographic and lifestyle factors such as age, body mass index (BMI), alcohol consumption, intake of seafood, coffee and smoking habits were collected by questionnaires (Table 1) [26,27].

**Table 1:** Characteristics of the men in the study groups

		***Greenland n *= 75**	***Warsaw n *= 99**	***Sweden n *= 78**	***Kharkiv n *= 86**	***All n *= 338**
**Age **(years)	median	30	30	46	26	32
	min-max	23–47	18–46	24–67	16–45	16–67
						
**BMI **(Kg/m^2^)	median	26	26	26	24	25
	min-max	19–38	12–58	22–37	19–36	12–58
						
**Alcohol **(drink/week)	median	2.0	3.5	n.a	2.5	3.00
	min-max	0–35	0–30		0.2–15	0–35
						
**Smoking **(ever)	%	87	49	40	82	68
**Seafood **(days/week)	median	2.0	1.0	n.a	4.0	2.0
	min-max	0–9.0	0–9.0		1.0–9.0	0–9.0
						
**Coffee **(cups/day)	median	3.0	2.0	n.a	2.0	2.0
	min-max	0–20	0–6.0		1.0–7.0	0–20
						
**Total testosterone **(nmol/l)	median	14	13	12	18	14
	min-max	3.2–75	6.5–23	4.2–28	8.4–32	3.2–32
						
**Estradiol **(nmol/l)	median	59	72	67	81	71
	min-max	31–88	45–296	25–155	33–144	25–296

This study included in total 338 blood samples taken from males from Greenland (75), Sweden (78), Warsaw (99) and Kharkiv (86). Venous blood samples were collected in 10 ml vacuum tubes and after centrifugation the serum was transferred to Nunc tubes and stored at -80°C until analyzed [27].

### 2. 2. Determination of CB-153 and p,p'-DDE in serum

Serum concentrations of CB-153 and *p,p'*-DDE were determined using GC-MS after solid phase extraction and on-column degradation of lipids. CB-153 and *p,p'-*DDE levels were adjusted for serum lipids analyzed by enzymatic methods [27,31]. Levels of detection, coefficients of variation (CV) and participation in quality control programs have been described in detail elsewhere [27]. All analysis of CB-153 and *p,p'*-DDE were performed at the Department of Occupational and Environmental Medicine, University of Lund, Sweden.

### 2. 3. AhR-CALUX assay

#### 2. 3.1. Sample preparation

The extraction of serum sample to obtain the fraction containing lipophilic POPs for AhR-CALUX activity measurements was performed at a certified laboratory, Le Centre de Toxicologie, Sainte Foy, Quebec, Canada. Serum samples (2 ml) were mixed with an aqueous solution of ammonium sulfate and ethanol (1:1) and then extracted with hexane. Extracts were concentrated and cleaned by elution through two columns containing Florisil. The details of extraction has been described elsewhere [32]. The extracts dissolved in 500 μl hexane were stored at -80°C until analyzed.

#### 2.3.2. Dissolving of samples

The serum extracts were thawed and evaporated to near dryness at 30°C under the gentle stream of nitrogen. The sample solvent, 10 μl DMSO: H_2_O (5:5, v/v), was added to each sample vial and stored overnight at room temperature. After giving the samples a quick spin (1000 rpm, 25 sec.), 500 μl α-minimal essential medium (α-MEM, GibcoBRL, UK) was added, mixed completely and transferred to two new test tubes (250 μl/tube) each containing 417 μl supplemented α-MEM (α-MEM plus 10% fetal calf serum (FCS, GibcoBRL, UK), 64 μg/ml garamycin (Schering-Plough, Brussels, Belgium)) with or without the 60 pM (EC_50_) (see 2.3.3) TCDD (98%, Cambridge Isotopes Laboratories Inc., USA), respectively. The final serum extract was equal to 150 μl serum per well (96- well plate) which was shown to be in the linear range of the AhR mediated luciferease activity (Bonefeld Jorgensen and Long, manuscript in prep.). All the processes were protected from light.

#### 2.3.3. AhR-CALUX bioassay

The stable transfected mouse hepatoma cell line Hepa1.12cR carrying the AhR-luciferase reporter gene (kindly provided by M.S. Denison (University of California, USA)), induces luciferase in an AhR-, time- and dose-dependent manner [23]. In each independent experiment, a dose-response of TCDD (dissolved in DMSO and subsequently diluted in the supplemented α-MEM) was performed at concentrations ranging from 2 × 10^-12 ^to 5 × 10^-9 ^M (see additional file 1: TCDD dose-response curve for AhR mediated luciferase activity). The maximum effect concentration was 1000 pM, and the half maximum effect concentration (EC_50(TCDD)_) was calculated to be 60 pM by fitting the dose-response data into a three parameter sigmoidal Hill curve using Sigma Plot (SPSS, Chicago, IL, USA). The minimal detection limit was 64 fg/well with an intra CV of 5–10% and an inter CV of 10–20%. The sample solvent controls (+/- EC_50(TCDD)_) consisted of sample solvent treated like the serum extract but without the extract, and the EC_50(TCDD) _was used as parallel positive control in each assay on each plate.

AhR agonistic effect (AhRag) was determined by exposure of the cells to serum extracts alone, and the competitive AhR effect (AhRcomp) was determined upon co-exposure with serum extract and 60 pM TCDD (EC_50_). The AhR-CALUX assay can be described shortly as follows: The Hepa1.12cR cells were seeded into sterile 96-well white CulturPlate™ (Packard Instruments) at 6 × 10^4 ^cells per well and cultured in supplemented α-MEM containing 400 μg/ml geneticin (G418, Sigma-Aldrich) at 37°C, 5% CO_2 _in 95% humidified air for 24 h, allowing cells to reach 90–100% confluence. Then media were removed and the cells were in parallel exposed to the serum extract, serum extract plus 60 pM TCDD, and sample solvent controls in a total volume of 100 μl per well in triplicate. After exposure for 4 h, cells were washed with phosphate-buffered saline (PBS, pH 7.4) followed by addition of cell lyses buffer. Luciferase activity and cell protein were determined as described [33]. The luciferase activity was expressed in relative light units per microgram protein (RLUs/μg protein). The average intra-sample CV was 11% and the inter CV of solvent control was 19%.

No cell toxicity on Hepa1.12cR cells was determined by the CellTiter 96 assay from Promega (Madison. WI, US) [33] after exposure to the tested serum extract.

The determinations of AhR activity was in good inter-lab precision, as determined from the results of interlaboratory comparison program (Second round of interlaboratory comparison of dioxin-like compounds in food using bioassay, Orebro, Sweden).

### 2. 4. Calculation and statistical analysis

In the independent assays the activity differences between the triple serum extract determinations and their respective solvent controls (% agonistic, % antagonistic and % additive/synergistic, Table 2) were evaluated using the Student t-test (Microsoft Excel).The data was given as RLU per ml serum and the value of the solvent controls was 6.67 RLU/ml serum.

**Table 2:** AhR-mediated serum activities, TCDD equivalents and lipid adjusted POP markers in serum

		**Greenland**	**Warsaw**	**Sweden**	**Kharkiv**	**All**
AhRag*^1 ^(RLU/ml serum)	N	75	99	78	86	338
	**Median**	**24**	**34**	**33**	**26**	**29**
	Min	6.6	11	8.0	8.8	6.6
	Max	257	118	103	56	257
	% agonist	92	100	95	100	97
AhR-TEQ*^2 ^(pg/g lipid)	N	70	99	76	80	325
	**Median**	**197**	**312**	**428**	**337**	**310**
	Min	38	72	104	110	38
	Max	1188	1054	1261	781	1261
AhRcomp*^3 ^(RLU/ml serum)	N	75	99	78	86	339
	**Median**	**8.3**	**6.4**	**6.2**	**5.5**	**6.8**
	Min	3.9	3.2	1.6	1.5	1.5
	Max	16	9.1	10	11	16
	% add/syn	41	3.0	6.4	18	16
	%antagonist	2.7	8.0	12	34	14
**CB-153 **(ng/g lipid)	N	74	100	98	82	355
	**Median**	**220**	**16**	**210**	**47**	**78**
	Min	5.1	3.3	41	5.5	3.3
	Max	5500	130	1500	200	5500
***p,p'*-DDE **(ng/g lipid)	N	74	100	98	82	355
	**Median**	**630**	**570**	**240**	**880**	**560**
	Min	66	240	55	320	55
	Max	13000	2100	2300	12000	13000

*^1^: AhRag: AhR activity of serum extract alone determined as the relative luciferase activity (RLU) per ml serum; Solvent background control = 6.67 RLU/ml serum. The % agonistic indicates the % of samples eliciting a significant increase in AhR activity compared to the solvent control. *^2 ^AhR-TEQ (TCDD equivalents): The samples eliciting significantly agonistic activity was calculated by interpolation to the TCDD dose-response curve using the Sigmaplot program, given as pg/g serum lipid. *^3 ^AhRcomp: AhR competitive activity of s*erum extract *+ 60 pM TCDD (EC_50_) given as RLU/ml serum. EC_50 _solvent control = 6.67 RLU/ml serum; % add/syn and %antagonistic indicates the % of samples responding with a further increase or decrease of the EC_50(TCDD) _induced activity, respectively.

The CALUX-based AhR-TEQs values of serum extract were obtained by interpolation of AhRag values onto the TCDD dose-response sigmodal Hill curve (see additional file 1: TCDD dose-response curve for AhR mediated luciferase activity). Only the AhRag values being significantly higher than the solvent control and in the linear range of the TCDD dose-response curve were used to calculate AhR-TEQ.

The natural logarithmic transformed AhR-mediated activities and markers of POPs improved the normality (checked by Q-Q plots) and homogeneity of variance, and the statistical analysis was performed on the ln-transformed data. The comparisons of means between the different variables (POP markers, AhRag, AhR-TEQ and AhRcomp) were performed with One-way ANOVA test. When ANOVA showed statistical significant difference complementary multiple comparison *ad hoc *tests was performed. Test for equal variances was performed with Levene's test. The least-significant difference (LSD) test was used if the variables have equal variance; otherwise Dunett T3 test was used.

Bivariate correlations were evaluated by Spearman's rank correlation test. The overall association between the POP markers and AhR-mediated activities across the study groups (combined data) was assessed by comparing the regression lines for each study group by using multiple regression analysis.

Up to date few studies on dioxin-like activities in human serum have been reported [34-37], and thus the knowledge is limited about which dietary or other life-style determinants might affect serum dioxin-like activity. Our hypothesis is that potential determinants of POP bioaccumulation might also be potential determinants for serum dioxin-like activity. Guided by the literature [38] and also from the assessment of the main Inuendo study populations [26,27], age and seafood are known determinants affecting the POP serum level. Moreover, lifestyle characteristics (Table 1) were evaluated as potential determinants of AhRag, AhR-TEQ and AhRcomp levels. Multiple linear regression model was used to assess the impact of the POP biomarkers on AhRag, AhR-TEQ and AhRcomp. The impact of potential confounders were evaluated by entering blocks of variables together with either CB-153 or *p,p'*-DDE as follows: in the first step, age and seafood intake were included in the model, and in the second step additionally smoking status, BMI, coffee intake and alcohol consumption were included in the model. Due to many missing values on the potential confounders the number of available observations in the confounder analyses are much smaller than in the unadjusted analysis on the full dataset (full dataset: n = 338, first step of confounders: n = 232, second step: n = 164). A reduction of the number of observations with more than 50% might introduce serious selection problems, and hence the confounder analyses might lack greater validity.

The AhR activities were tested in protein free serum extract, which may still contain endogenous steroids, thus testosterone (total and free) and estradiol [39] were further included in the linear regression model on the combined study group data.

The inter-population variations in POP markers, AhRag, AhR-TEQ and AhRcomp serum level were also assessed by linear regression models. In these models age was considered as a potential confounder to make age-adjusted comparisons.

The statistical analysis was performed on SPSS 13.0 (SPSS Inc. Chicago, IL, USA). The statistical significant level was set to p ≤ 0.05.

## 3. Results

### 3.1. The basic characteristics and serum levels of CB-153 and p,p'-DDE

The distributions of demographic and lifestyle factors (Table 1) and the serum CB-153 and *p,p'*-DDE levels (Table 2) of the 338 adult males in this study were similar to that of the main Inuendo study population [26,27]. The Greenland and Swedish groups elicited higher CB-153 level than the Kharkiv and Warsaw group, and the level of CB-153 of Warsaw group was significantly lower than the other groups (Table 2 and see additional file 2A: Multiple comparisons of variables). The *p,p'*-DDE level in Kharkiv was the highest, followed by Greenland and Warsaw, and the significant lowest level was found for the Swedish fishermen (Table 2 and see additional file 2A: Multiple comparisons of variables). As for the main study [27], age adjustment did not change the pattern of difference for the POP markers between the study groups.

A higher correlation between serum concentration of CB-153 and *p,p'*-DDE was found for the Greenlandic (r_s _= 0.94, p < 0.001) and Swedish groups (r_s _= 0.75, p < 0.001), and lower correlations were observed for the Kharkiv (r_s _= 0.45, p < 0.001) and the Warsaw (r_s _= 0.27, p < 0.01) study groups (see additional file 2B: Spearman's correlation between serum AhR activities and the levels of CB-153 and *p.p'*-DDE).

### 3.2. Agonistic and competitive AhR activity in the four study groups

**Figure 1:**
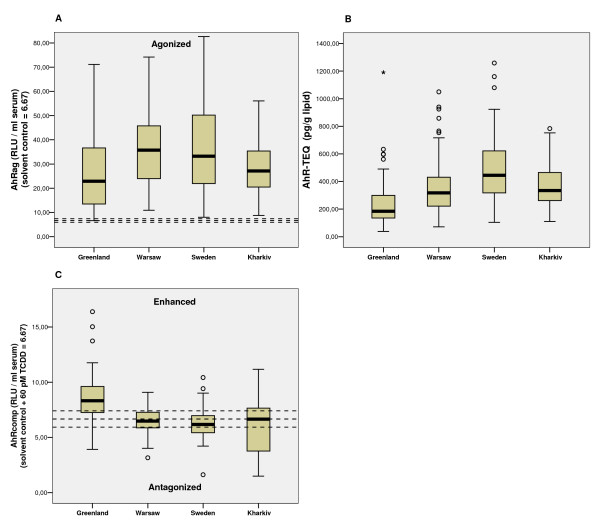
**AhR-CALUX activities of the study groups**. (A) Agonistic activity of serum extracts alone (AhRag), (B) AhR-TEQ (AhR-CALUX- TCDD toxic equivalent) and (C) competitive AhR activity upon cotreatment with 60pMTCDD (EC_50_) and serum extract (AhRcomp). For the AhRag the outliers ranging from 86.01 to 111.28 RLU/ml serum and extreme values (117.87 – 257.13 RLU/ml serum) are not shown. The reference lines of the respective solvent controls ± SD (6.67 ± 0.74) are given as dashed lines.

Almost all serum samples (97%) showed AhRag activity significantly higher than the solvent control (Table 2). The AhRag activity and AhR-TEQ differed significantly among the study groups (Table 2 and see additional file 2A: Multiple comparisons of variables): the Greenland and Kharkiv groups had lower AhRag medians than Sweden and Warsaw groups (Fig. 1A and see additional file 2A: Multiple comparison of variables), and the AhR-TEQ median level of Greenland group was significantly lower than that of the European study groups (Table 2, Fig 1B and see additional file 2A: Multiple comparisons of variables).

The Greenlandic AhRcomp activity, eliciting the highest incidence (41%) of sample with further increasing TCDD induced AhR activity, was significantly higher than for the European groups (Table 2, Fig. 1C and see additional file 2A: Multiple comparisons of variables), whereas the Kharkiv group showed the highest frequency of sample with antagonistic effect on TCDD induced AhR activity (Table 2).

Compared to the crude data, the pattern of differences of AhRag, AhR-TEQ and AhRcomp between the study groups did not change after adjustment for age (data not shown).

### 3.3. Associations between AhRag, AhR-TEQ, AhRcomp and the POP markers

**Figure 2:**
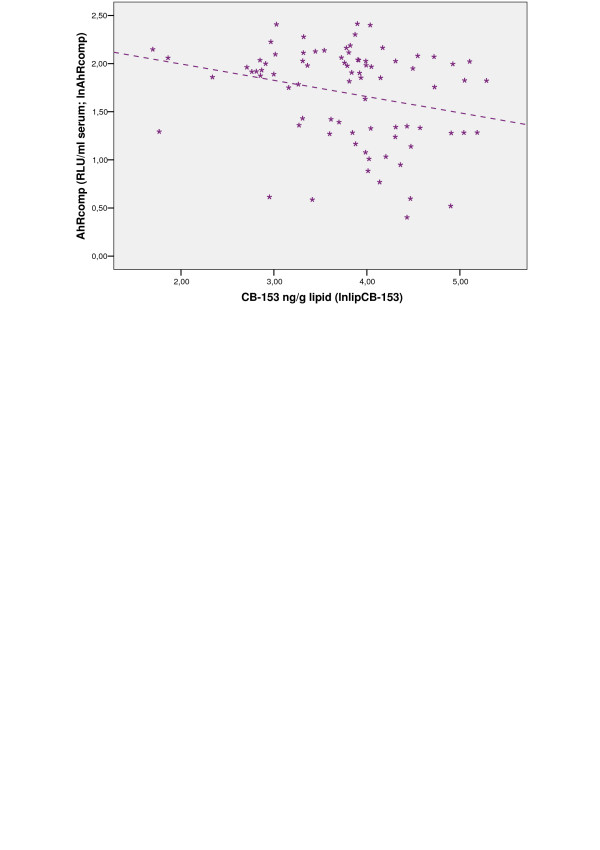
**The relationship between AhRcomp activity and CB-153 for the Kharkiv group**. Scatterplot of correlation between serum AhRcomp and CB-153. For definitions of AhRcomp see legend to Table 2. Ln-transformed data was used. RLU: relative luciferase units.

Significant inverse correlation (r_s _= -0.30, p < 0.01) between AhRcomp and CB-153 was observed for the Kharkiv group (Fig. 2 and see additional file 2B: Spearman's correlation between serum AhR activities and the levels of CB-153 and *p.p'*-DDE). Neither AhRag nor AhR-TEQ was found to correlate to the two POP markers for any of the study groups (see additional file 2B: Spearman's correlation between serum AhR activities and the levels of CB-153 and *p.p'*-DDE).

Adjustment for potential confounders in the multiple regression models did not change the strength of association between exposure variables (POP markers) and outcome variables (AhR-mediate activities) when compared with the unadjusted models. Further adjustment for endogenous testosterone and estradiol neither changed this pattern (data not shown).

### 3. 4. Multiple regressions of AhR-mediated activities on POP markers across the study groups

**Figure 3:**
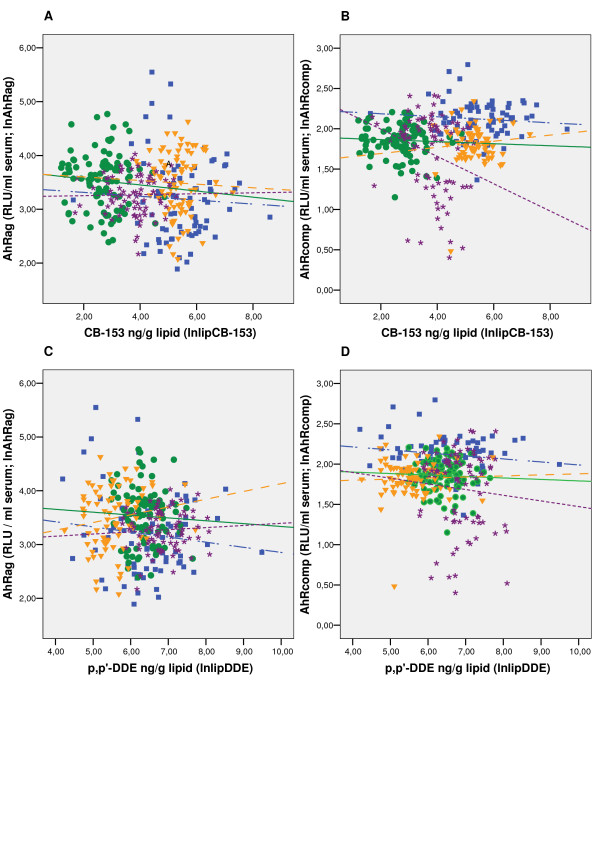
**The association between AhR activity and POP markers in the study groups**. The AhR activities are given for the four country based study groups as relation between (A) AhRag and CB-153, (B) AhRcomp and CB-153, (C) AhRag and *p,p'*-DDE, (D) AhRcomp and *p,p'*-DDE. Ln-transformed data was used. For the definition of AhRag and AhRcomp, see the legend to Table 2. RLU: relative luciferase units.

Scatter plots of AhR-mediated activities against POP markers for the study groups are shown in Figure 3. Multiple regression analysis showed homogeneity of associations between CB-153 or *p,p' *-DDE and AhRag and AhR-TEQ as well as AhRcomp and *p,p' *-DDE across the study groups (Table 3), i.e. parallel regression lines among study groups. Furthermore, a model with parallel regression lines showed a significant differences between the intercepts of the study groups (Table 3), thus the differences in AhRag/AhR-TEQ/AhRcomp between study groups still exist after adjustment for CB-153 or *p,p' *-DDE. However, heterogenetic associations between serum CB-153 and the AhRcomp across the study groups were found (Table 3). Thus the difference of AhRcomp between the study groups was correlated to the CB-153 level, which supports the negative correlation between AhRcomp and CB-153 for the Kharkiv group.

**Table 3:** Multiple regressions of the combined study groups

Response variable	Homogeneity of slope (p value)	Common slope, Estimate (SE), p value	Common intercept (p value)	Adjusted R square
**AhRag (n = 327)**				
CB-153	0.95	-0.03 (0.04), 0.43	**0.001**	0.05
*p,p'*-DDE	0.28	-0.02 (0.05), 0.70	**0.001**	0.05
				
**AhR-TEQ (n = 324)**				
CB-153	0.54	0.11(0.04), 0.77	**< 0.001**	0.20
*p,p'*-DDE	0.99	0.08(0.05), 0.07	**< 0.001**	0.20
				
**AhRcomp (n = 327)**				
CB-153	**0.01**	-*	-*	-*
*p,p'*-DDE	0.80	-0.03 (0.03), 0.24	**< 0.001**	0.18

Both AhR activities and POP markers are ln transformed. Homogeneity of slope: test for homogeneity of association between exposure variables and outcome variables across the study group (p > 0.05, accept the hypotheses of homogeneity of slope). Common slope: the estimated common slope across study groups assuming homogeneity (p > 0.05, accept the hypotheses that slope equals to zero). Common intercept: test of a common intercept across study groups assuming a common slope (p > 0.05, accept the hypotheses having common intercept across the study groups). Adjusted R square assumes a common slope. *: Since heterogeneity of slope exists between CB-153 and AhRcomp across the study groups, i.e. there were country differences in the association of CB-153 and AhRcomp, no further evaluation was performed.

## 4. Discussion

In the present study we measured the integrated AhR mediated activity in the lipophilic serum fraction containing POPs using the mechanistically based AhR-CALUX bioassay. No consistent correlation between the POP markers (CB-153 and *p,p'*-DDE) and AhR mediated activities were found. However, in accordance with its high incidence of antagonistic AhRcomp activity, a negative correlation between AhRcomp and CB-153 was found in Kharkiv group. This finding is supported by earlier reports that CB-153 and associated compounds antagonized TCDD induced AhR action [40,41], as well as observation in our laboratory (Long and Bonefeld-Jorgensen, manuscript in prep.).

The study showed that 97% of the serum samples across the study groups elicited significant agonistic AhR activity. The median of AhRag activity in the Warsaw and Swedish groups reached higher level compared to the Kharkiv and the Greenlandic groups. Moreover, the median levels of AhR-TEQ of the European study groups were significantly higher than that of the Greenlandic Inuit's having high burden of both CB-153 and *p,p'*-DDE. We do not exactly know the dietary habit of the Warsaw study group. However, relatively high level of dioxins and DLCs were reported to be produced and emitted to the Polish environment [42,43]. The relatively higher AhRag and AhR-TEQ in the Swedish study group may be caused by high exposure to dioxins and/or dioxin-like PCBs (DL-PCBs) [8].

Except for a recent study of Slovakia males [44], the AhR-TEQ in the present study was relatively higher (2~8 fold) than other similar studies [35,37], which can be related to the difference in exposure time (4 hours versus 24 hours). During the optimal exposure time (4 h) of Hepa1.12cR cell line used in this study [45,46], other active compounds in the lipophilic serum fraction such as polycyclic aromatic hydrocarbons (PAHs), polybrominated biphenyls (PBBs), polyhalogenated naphthalenes may interact and activate the AhR in the CALUX- bioassay. In other studies e.g. using rat H4IIE cells, the labile AhR agonists in the serum such as some PAHs and other chemicals may be degraded or metabolized during the 24 hours of exposure resulting in a relatively lower AhR response [47]. PAHs elicite high AhR response after short exposure [47,48], nevertheless, in the present study the PAHs level would be very low in the serum extract because the purification method was not built specifically for these molecules. We do not assume that the endogenous compounds play a major role in our AhR response data because fatty acids and other nonclassical AhR ligands may be removed from the crude extract in the cleanup procedure [24,32]. However, we can not exclude that some organic endogenous AhR inducers possibly passed through the purification columns and contribute to the AhR response [49]. Owing to the possible existence of cross-talk between AhR and ER and AR, it can not be excluded whether the xenohormones and/or endogenous sex hormones (estradiol and testosterone) influence the observed AhR response. Although the influence is expected to be of minor importance since no correlation between the sex hormones and AhR-mediated activity across the study groups were found. The extract in this study contains most organochlorine compounds including organochlorine pesticides like hexachlorobenzene (HCB) that can contribute to the AhR response [50]. Future investigations are required to elucidate the profile of serum compounds contributing to the AhR-CALUX response. Possibly, the gender might also influence the lipid adjusted AhR-TEQ level since the participants in other studies were female [35,36].

The differences in AhRag/AhRcomp activities among the study groups suggest that there are regional differences in profiles of POPs, PAHs and/or other lipophilic AhR activating compounds. The higher frequency of samples further increasing the TCDD induced AhR activity in Inuit's indicated the presence of compounds which can enhance the effect of the TCDD, the most potent AhR ligand. Considering the further increase of AhRcomp using the TCDD dose-response curve (see additional file 2: TCDD dose-response curve for AhR mediated luciferase activity), the median AhR-TEQ determined in serum of the corresponding subgroup (n = 180) was calculated to be 1.3 ng/g lipid, suggesting an increasing risk from DLCs when a strong AhR ligand exists simultaneously in the body.

Studies of Inuit populations in Canada support in general the use of CB-153 as a surrogate marker of exposure to non-DL-PCBs present in the Arctic food-chain [51]. However, the level of coplanar and *non-coplanar *PCBs was shown to differ between Canadian Inuits and Caucasian [32,52]. For non-occupational exposed Inuit populations in the Arctic Quebec the ratio between coplanar PCBs (e.g. CB-126 or CB-169) and the *non-coplanar *PCB (e.g. CB-153) of Inuits was lower than that of the Caucasian reference group [52]. Moreover, DL-PCBs contributed to a larger extent to the chemical calculated TEQ than PCDD and PCDFs, with the *mono-ortho *coplanar CB-118 as major contributor for the total toxicity [52]. Similar difference may also exist for the populations included in this study, supporting the higher AhRag or AhR-TEQ level of Europeans.

Previously, CB-153 was reported to be highly correlated with calculated chemical-derived total TEQs and/or PCDD/PCDFs TEQs and/or *non-ortho *PCBs TEQs [29]. It should be noted that the chemical derived TEQ was calculated according to analytical chemistry data of some congeners under the assumption of additivity. Even though additive effects of PCDDs/PCDFs/DL-PCBs are predominating, non-additive effects such as antagonism and synergism can apply to interactions between individual DLCs in a complex mixture [53]. The TEQ based on the AhR-CALUX bioassay represents the integrated sum of dioxin-like activities including additive, synergistic and antagonistic effect. Few epidemiology studies have reported analysis of association between *di-ortho *PCB (including CB-153) and CALUX-TEQ, and the results were contradictory. A positive correlation was reported by Pauwels et al [35]. However, a recent report of the potential of the CALUX bioassay to estimate TEQ in plasma of Italian women with background exposure to dioxin and DLCs showed no significant correlation between CALUX-TEQ and the sum of four major PCB congeners (CB-118, CB-138, CB-153, CB-180) [36]. Similar result was reported in a Belgium study [34]. The contradictory results may be related to the differences in study design, sample selection (gender and age of subjects, serum or plasma), composition and concentrations of bio-accumulated compounds in the blood and/or different sample extraction methods used in the CALUX bioassay as discussed [36,49,54]. Moreover, an important part of dioxin-like activity is elicited especially by PCDD/Fs when their concentration is high in the sample [55]. One can find some correlations between PCB levels and dioxin-like activity only in the case that the concentrations of PCDD/Fs are low or comparable in different cohorts [34,56].

## 5. Conclusion

97% of analyzed samples elicited significant agonistic AhR induced activity. The level of AhR mediated activities differed among the study groups. European groups elicited higher AhR-TEQ than the Greenland Inuits, suggesting a higher exposure to DLCs. In addition, dietary habits/life style factors and the genetic difference between Inuits and Caucasians [57] may also be taken into account.

No consistent significant correlation between CB-153 and *p,p'*-DDE and AhR activities was observed and these two selected POP markers cannot alone predict the contribution of POPs, PAHs, and other lipophilic xenobitics to serum dioxin-like activity. Other more sensitive and specific tentative markers such as DL-PCBs (e.g. the mono-ortho congener CB-118 and CB-156) should be included in future epidemiology studies.

Since some less persistent compounds might contribute to the AhR-CALUX response due to the selected fractionation technique and that *in vitro *AhR-CALUX detects the overall dioxin-like response, it is not clear which compounds contribute to the observed dioxin-like activity. Thus the serum dioxin-like activity determined in this study must be interpreted as an independent parameter, complementary to chemical data. It should be kept in mind that the AhR-CALUX bioassay is not a substitute of actual chemical analysis by GC-MS techniques but provide biologically relevant results and normally is utilized as first tier screening tool followed by the chemical analysis to identify specific response compounds [24]. AhR-CALUX bioassay provides an overall biological response/potency of mixture, whereas chemical analysis provides the concentration of specific compounds in the mixture.

## Abbreviations

**PCDDs/PCDFs **polychlorinated dibenzo-p-dioxins/furans

**PCBs **polychlorinated biphenyls

**DDT **2,2-bis(p-chlorophenyl)-1,1,1-trichloroethane

**POPs **persistent organochlorine pollutants

**CB-153 **2,2',4,4',5,5'-hexachlorobiphenyl

***p,p'*-DDE **1,1-dichloro-2.2-bis (p-chlorophenyl)-ethylene

**TCDD **2,3,7,8-tetrachlorodibenzo-p-dioxin

**AhR **aryl hydrocarbon receptor

**TEQs **TCDD toxic equivalents

**CALUX **Chemical activated luciferase gene expression

**TEFs **Toxic Equivalency Factors

**DLCs **Dioxin-like compounds

**DL-PCBs **dioxin-like PCBs

**GC-MS **gas chromatography mass spectrometry

**AhRag **agonistic AhR activity

**AhRcomp **competitive AhR activity

## Competing interests

The author(s) declare that they have no competing interest

## Authors' contributions

ML and ECB-J drafted the work and were the main responsible for design, performance, data evaluation and statistical analyses of the specific project; ML and BSA performed the mechanistic work; CL performed POP determinations in blood; JPB was main responsible for raising funding for the project. GT, HSP, VZ have been responsible for collecting blood samples and for obtaining the interview data. JPB, AG and LH initiated and designed the Inuedo project. JPB and GT coordinated the execution of project and GT had main responsibility for creating the joint database. All authors participated in the design of the study, comment on the draft and have read and approved the final manuscript.

## Supplementary Material

Additional file 1 **TCDD dose-response curve for AhR mediated luciferase activity**. The 96-well plates containing Hepa1.12cR cells at 90–100% confluence were incubated with TCDD at the indicated concentration for 4 hours. Luciferase activity in cell lysates were determined and corrected to cell protein. The data is expressed as the luciferase activity above solvent control which was set to 1. Values represent the mean ± S.D. (n ≥ 5).The EC_50 _is the half maximum effect concentration. Click here for file

Additional file 2A **Multiple comparisons of variables**. Multiple comparisons were performed on ln-transformed data. The values given are p values. **B** Spearman's correlation between serum AhR activities and the levels of CB-153 and *p,p' *-DDE. Continuous data was used. Spearman's correlation data is given. For definition of AhRag, AhRcomp and AhR-TEQ see legend to Table 2. Statistical significant data is given in bold. Click here for file
