# Supplementary material for: Dioxin-like activities in serum across European and Inuit populations
Source: Environ Health. 2006 May 25;5:14. doi: 10.1186/1476-069X-5-14 (PMC1501006; doi:10.1186/1476-069X-5-14)
Supplement: Additional file 2A — Multiple comparisons of variables. Multiple comparisons were performed on ln-transformed data. The values given are p values. B Spearman's correlation between serum AhR activities and the levels of CB-153 and p,p' -DDE. Continuous data was used. Spearman's correlation data is given. For definition of AhRag, AhRcomp and AhR-TEQ see legend to Table 2. Statistical significant data is given in bold. [file 1476-069X-5-14-S2.doc]

**Additional file 2A: Multiple comparisons of variables**

Multiple comparisons were performed on ln-transformed data. The values given are p values.

|  |  | ***All study group data*** | **Greenland (GR, sum)** | **Sweden (SE)** | **Kharkiv (UA)** |
| --- | --- | --- | --- | --- | --- |
| **AhR ag** |  | **<0.001** |  |  |  |
|  | **Warsaw** |  | **0.004** | 0.99 | **0.001** |
|  | **Sweden** |  | **0.03** | - | 0.07 |
|  | **Kharkiv** |  | 0.86 |  |  |
| **AhR-TEQ** |  | **<0.001** |  |  |  |
|  | **Warsaw** |  | **<0.001** | **<0.001** | 0.30 |
|  | **Sweden** |  | **<0.001** | - | **0.004** |
|  | **Kharkiv** |  | **<0.001** |  |  |
| **AhRcomp** |  | **<0.001** |  |  |  |
|  | **Warsaw** |  | **<0.001** | 0.15 | 0.65 |
|  | **Sweden** |  | **<0.001** | - | 0.67 |
|  | **Kharkiv** |  | **<0.001** |  |  |
| **CB-153** |  | **<0.001** |  |  |  |
|  | **Warsaw** |  | **<0.001** | **<0.001** | **<0.001** |
|  | **Sweden** |  | 0.611 | - | **<0.001** |
|  | **Kharkiv** |  | **<0.001** |  | - |
| ***p,p’*-DDE** |  | **<0.001** |  |  |  |
|  | **Warsaw** |  | 0.941 | **<0.001** | **<0.001** |
|  | **Sweden** |  | **<0.001** | - | **<0.001** |
|  | **Kharkiv** |  | **<0.001** |  |  |

**Additional file 2B: Spearman’s correlation between serum AhR activities and the levels of CB-153**

**and *p,p’*-DDE**

|  |  | **Greenland** | | | **Warsaw** | | | **Sweden** | | | **Kharkiv** | | | |
| --- | --- | --- | --- | --- | --- | --- | --- | --- | --- | --- | --- | --- | --- | --- |
|  |  | *n* | r_s_ | p | *n* | r_s_ | p | *n* | r_s_ | p | | *n* | r_s_ | p |
|  | **CB-153** |  |  |  |  |  |  |  |  |  | |  |  |  |
|  | AhRag | 73 | .06 | .64 | 99 | -.08 | .45 | 76 | .01 | .93 | | 80 | .01 | .94 |
|  | AhR-TEQ | 70 | .14 | .25 | 99 | -.10 | .32 | 76 | -.05 | .69 | | 80 | .08 | .48 |
|  | AhRcomp | 73 | -.03 | .83 | 99 | .05 | .66 | 76 | -.06 | .62 | | 80 | **-.30** | **<.01** |
|  |  |  |  |  |  |  |  |  |  |  | |  |  |  |
|  | ***p,p’*-DDE** |  |  |  |  |  |  |  |  |  | |  |  |  |
|  | AhRag | 73 | .03 | .83 | 99 | -.06 | .56 | 76 | .16 | .16 | | 80 | .03 | .82 |
|  | AhR-TEQ | 70 | .15 | .21 | 99 | .07 | .51 | 76 | .07 | .54 | | 80 | .12 | .28 |
|  | AhRcomp | 73 | -.09 | .46 | 99 | .03 | .77 | 76 | -.06 | .63 | | 80 | -.06 | .58 |
|  |  |  |  |  |  |  |  |  |  |  | |  |  |  |
| CB-153/*p,p’*-DDE | | 74 | **.94** | **<.001** | 100 | **.27** | **<.01** | 98 | **.75** | **<.001** | | 82 | **.45** | **<.001** |

Continuous data was used. Spearman’s correlation data is given. For definition of AhRag, AhRcomp and

AhR-TEQ see legend to Table 2. Statistical significant data is given in bold.
