# Supplementary material for: Dioxin-like activities in serum across European and Inuit populations
Source: Environ Health. 2006 May 25;5:14. doi: 10.1186/1476-069X-5-14 (PMC1501006; doi:10.1186/1476-069X-5-14)
Supplement: Supplementary file 2 [file 1476-069X-5-14-S1.doc]

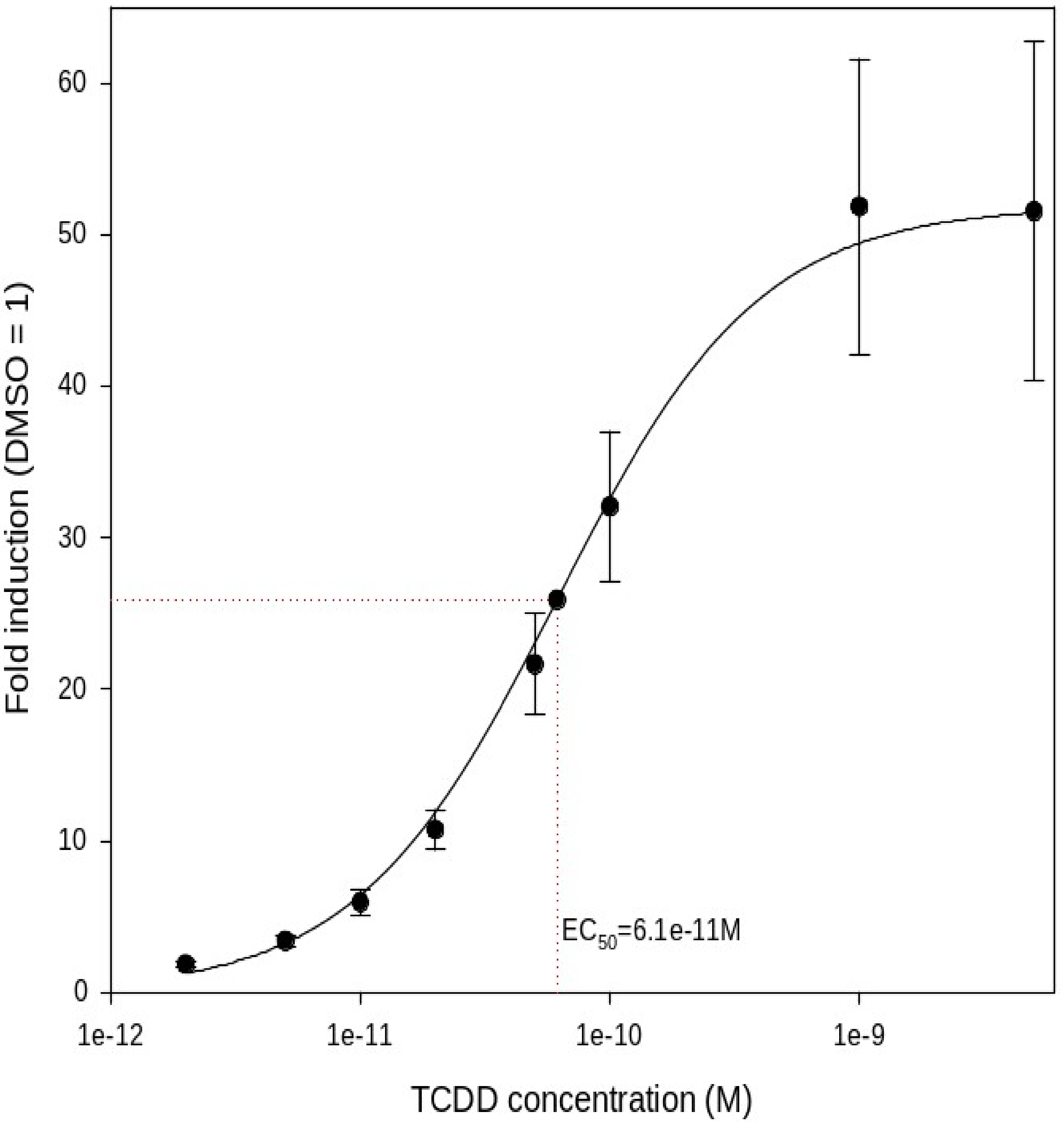
Fold induction (DMSO = 1)
0
10
20
30
40
50
60
EC$_{50}$=6.1e-11M
1e-12
1e-11
1e-10
1e-9
TCDD concentration (M)
